# Construct social‐behavioral association network to study management impact on waterbirds community ecology using digital video recording cameras

**DOI:** 10.1002/ece3.7200

**Published:** 2021-02-01

**Authors:** Muhammad Awais Rasool, Xiaobo Zhang, Muhammad Azher Hassan, Tanveer Hussain, Cai Lu, Qing Zeng, Boyong Peng, Li Wen, Guangchun Lei

**Affiliations:** ^1^ School of Ecology and Nature Conservation Beijing Forestry University Beijing China; ^2^ Center for East Asian‐Australasian Flyway Studies Beijing Forestry University Beijing China; ^3^ Key Lab of Indoor Air Environment Quality Control School of Environmental Science and Engineering Tianjin University Tianjin China; ^4^ Department of Forestry, Range and Wildlife Management The Islamia University of Bahawalpur Bahawalpur Pakistan; ^5^ West Dongting Lake National Nature Reserve Hunan China; ^6^ Science Division NSW Department of Planning, Industry and Environment Sydney NSW Australia

**Keywords:** behavior interaction preferences, community ecology, species interaction preferences, video recording cameras, wintering habitat selection

## Abstract

Studying social‐behavior and species associations in ecological communities is challenging because it is difficult to observe the interactions in the field. Animal behavior is especially difficult to observe when selection of habitat and activities are linked to energy costs of long‐distance movement. Migrating communities tend to be resource specific and prefer environments that offer more suitability for coexisting in a shared space and time. Given the recent advances in digital technologies, digital video recording systems are gaining popularity in wildlife research and management. We used digital video recording cameras to study social interactions and species–habitat linkages for wintering waterbirds communities in shared habitats. Examining over 8,640 hr of video footages, we built tetrapartite social‐behavioral association network of wintering waterbirds over habitat (*n* = 5) selection events in sites with distinct management regimes. We analyzed these networks to identify hub species and species role in activity persistence, and to explore the effects of hydrological regime on these network characteristics. Although the differences in network attributes were not significant at treatment level (*p* = .297) in terms of network composition and keystone species composition, our results indicated that network attributes were significantly different (*p* = .000, *r* ^2^ = .278) at habitat level. There were evidences suggesting that the habitat quality was better at the managed sites, where the formed networks had more species, more network nodes and edges, higher edge density, and stronger intra‐ and inter‐species interactions. In addition, we also calculated the species interaction preference scores (SIPS) and behavioral interaction preference scores (BIPS) of each network. The results showed that species synchronize activities in shared space for temporal niche partitioning in order to avoid or minimize any potential competition for shared space. Our social network analysis (SNA) approach is likely to provide a practical use for ecosystem management and biodiversity conservation.

## Graphical abstract

Studying social‐behavior and species associations in ecological communities is challenging. We used digital video recording cameras to study social interactions and species–habitat linkages for wintering waterbirds communities in shared habitats. We built tetrapartite social‐behavioral association network of wintering waterbirds over habitat (*n* = 5) selection events in sites with distinct management regimes. We analyzed these networks to explore the regime effect and to identify hub species and species role in activity persistence. We found that habitats were significantly different (*p* = .000, *r* ^2^ =.278) in network composition and keystone species composition. Our SNA approach is likely to provide a practical use for ecosystem management and biodiversity conservation, where there is a need to make decisions on.

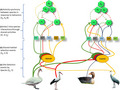

## 1 INTRODUCTION

Habitat selection and resource exploitation behaviors serve to understand the fitness and suitability effects of the prevailing environment, especially for migratory animal species. Migratory animals tend to be resource specific for selection of habitat for any given activity (Cody, 1985). The repeated activity‐based habitat selection events, therefore, provide information on habitat quality and use preferences (Farine et al., 2015; Pike et al., 2008). Species distributions and abundance estimates are important indicators of the health of ecosystems. They often correspond to environmental and human‐induced pressures on an ecosystem, as activity‐based habitat exploitation typically influences species distributions (Shettleworth, 2012), gives rise to inter‐specific competition for resources (Laird & Schamp, 2008), skews species diversity toward suitable habitat patches (Stonehouse et al., 2015), and maintains species abundance for healthy and social populations (Farine et al., 2015; Sadovy, 2001).

Human exploitation and anthropogenic alterations in many cases resulted in wildlife habitat deterioration for fitness and suitability (Aharon‐Rotman et al., 2017; Schmieder et al., 2006; Wang et al., 2017). These altered habitats are less likely to be occupied and used by animal species if compared to unaltered natural habitats. Management of the components (e.g., hydrology or vegetation) of these habitats has great implications for resulting species composition and their habitat use. Species richness and community composition, therefore, during each habitat selection and use trajectory, may result in complex interactions between co‐occurring species (Bridges & Noss, 2011; Crowder & Cooper, 1982; Wilman et al., 2014). In heterogenous habitats, resulting species community may comprise members from a broad trophic guild. Resource use in such communities depends upon the facilitative interactions between the species, forming a network of behavioral communication within the community (Croft et al., 2005; Crowder & Cooper, 1982; Rosenthal et al., 2015).

Ecological networks are ubiquitous (Montoya et al., 2006). Social network analysis (SNA) has been recently introduced into community ecology and has progressed rapidly over the last decade, especially in conjunction with the ability to collect, handle, and analyze large datasets (Farine & Whitehead, 2015). SNA has focused on the complex web of relationships found in animal groups and populations(Croft et al., 2008; Krause et al., 2015; Wey et al., 2008) to identify the role that particular individuals or species play in their social systems (Krause et al., 2010; Lusseau & Newman, 2004; Snijders et al., 2017). The questions arise here are whether and how species are consistent in occupying certain positions (i.e., keystones or hubs) within their network. The existence of fine‐scale social structures in communities or species groups has led to reconsideration of major topics in community ecology including population dynamics and habitat management (Dubois et al., 2016; Halley & Rosell, 2002), connecting biodiversity to ecosystem functioning (Creamer et al., 2016), identifying keystone species (Borrett, 2013; Zhao et al., 2016), animal behavior (Croft et al., 2004; Fletcher et al., 2013; Krause et al., 2003; Sih et al., 2009) and animal movements (Bridges & Noss, 2011; Ledee et al., 2016).

The activity pattern and synchronization in social communities strongly depend upon the interacting species in shared space and time. However, quantifying interactions for the whole ecosystem is costly (Hagen et al., 2012; Lindeman, 1942; Odum, 1957) because the large number of interactions between individuals or species pairs in a population may easily become intractable, presenting a key restriction in the studies of social interactions at the community scale. This barrier has led scientists to study interactions within small subsets of closely related species (e.g., trophic guilds) and to use dimensionality reduction based on multivariate, correlative approaches (Cumming et al., 2012; Mukaka, 2012) in community ecology.

We construct a social‐behavioral association network (SBAN) of species, from behavior‐based species abundance data, using video records. SBAN is “a network of species in a community where species are interconnected with other co‐occurring through behavioral communication, principally to exploit shared resources (Box 1).” The proposed approach offers a basis to study how the activities as a network are linked to habitat selection events and how behavioral niche partitioning between species minimize the potential competition among co‐occurring communities. We focused 14 wintering migratory waterbirds species that belong to five functional groups according to their feeding habits (Cumming et al., 2012; Del Hoyo et al., 1992; Table 1). We then frame a utility matrix on habitat selection and use preferences under two hydrological conditions (i.e., managed [R1] and unmanaged [R2]) for restored and natural lakes respectively. Using the utility matrix on two wintering seasons video record data (2016–17 and 2017–18), we aim to address two key research questions:

**TABLE 1:** Foraging‐based description of wintering birds’ functional groups in West Dongting Lake National Nature Reserve

Foraging guild	Species included
Fish, clam and invertebrate Eater (G1)	*Ciconia nigra, Ardea alba, Ardea cinerea, Ciconia boyciana, Anser cygnoides*
Tuber Feeder (G2)	*Grus leucogeranus, Grus monacha, Cygnus columbianus*
Sedge/Grass Forager (G3)	*Grus grus, Anser albifrons, Anser fabalis*
Invertebrate Eater (G4)	*Platalea leucorodia*
Fish Eater (G5)	*Larus* spp., *Pelecanus onocrotalus*

Box 1 **Important definitions used in this study**

**Table:** 

Term	Definition
Social‐behavioral association network	Social‐behavioral association network (SBAN) is a network of species in a community where species are interconnected with other co‐occurring through behavioral communication, principally to exploit shared resources.
Keystone species	A species that plays a crucial role in the way an ecosystem maintains its integrity. Disturbance to keystone species alters the ecosystem structure to degradation in long run.
Hub centrality	The capacity of a node (species) to influence and mediate between other nodes (peripheral species) by its virtual connectivity.
Activity	Key behavior for habitat selection and essential for species survival at wintering grounds (i.e., foraging and roosting).
Behavior	A short‐term inter‐ and intra‐species interaction (i.e., aggression, competition and courtship) that may occur while performing resource exploitative activities (i.e., foraging or roosting).
Species interaction preference scores (SIPS)	Quantitative measure of any intra‐ or inter‐species interactions during shared activity.
Behavioral interaction preference scores (BIPS)	Quantitative measure of intra‐ or inter‐species‐specific behavioral interactions on how the species interacted
Activity synchrony	The spatio‐temporal facilitation behavior between co‐occurring species to exploit shared resources in principally to avoid any potential event for competition.

1. How the control regime affects the habitat selection in species with different functional groups?
2. How the species of different functional groups respond to each other in shared environment?

## 2 MATERIALS AND METHODS

### 2.1 Study area

**FIGURE 1:**
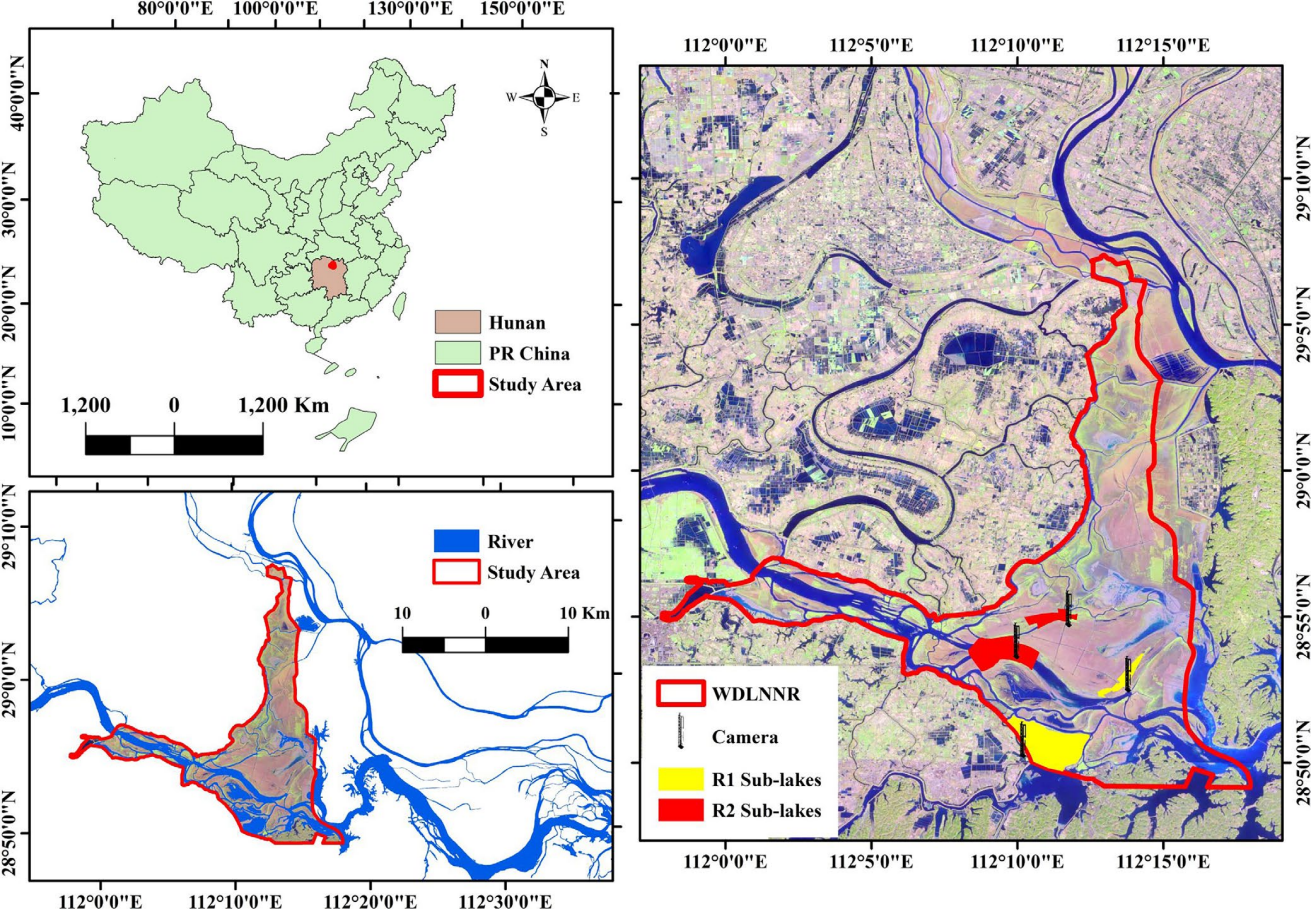
Study area map with location of managed (R1) and unmanaged (R2) sub‐lakes

West Dongting Lake National Nature Reserve (WDLNNR: 28°48′–29°07′N, 111°57′–112°19′E) is a Ramsar site and important wintering ground for many waterbirds species in the East Asian‐Australasian Flyway (EAAF). The lake has subtropical monsoon climate with a distinct wet/dry season where mean annual precipitation ranges from 1,200 to 1,415 mm. The average annual temperature ranges from 16.2 to 17.8°C with 259–277 frost‐free days. The elevation of the lake ranges from 27 to 30 m. During wet season (July–September), the majority of the lake is inundated with flood when water level rises from 20.13 m to 30.32 m. During dry season (November–February) as the water level drops, an array of sub‐lakes naturally converts to five main types of habitats, namely grassland, wet meadows, mudflats, shallow water and deep water (Lai et al., 2013; Li et al., 2014; Lu, Jia, et al., 2018) (Figure 1).

Since 1970, the Lake has undergone dramatic anthropogenic changes such as alteration of hydrological regimes and reclamation (Lu, Jia, et al., 2018; Zhang et al., 2016), and large area was converted to Poplar (*Populus* spp.) and common reed (*Phragmites australis*) plantation. These changes have led to sharp reduction in migratory waterbirds species richness and abundance (Li et al., 2014; Lu, Shi, et al., 2018; Zhang et al., 2016). Therefore in 2015, the Chinese government started a restoration program, which aims to sustain suitable habitats in some sub‐lakes through managing water level within the premises of WDLNNR. In the restored lakes (referred as R1 thereafter), controlled water level is managed to allow the development of permanent mudflats, grasslands, shallow water and open deep‐water habitats during the wintering period. In the unmanaged sub‐lakes (referred as R2 thereafter), the inundation regimes are determined by the hydrology of collected streams and local rainfall and are highly unpredictable and the water depth fluctuate in matter of hours and days.

### 2.2 Data collection

#### 2.2.1 Study site selection and deployment of digital video recording cameras

We considered waterbirds presence, human disturbance, and accessibility as key factors for study site selection. Primarily, we surveyed seven sub‐lakes during mid‐September 2016 to mid‐October 2016 and designated four best suitable lakes (2 restored and 2 reference lakes, Figure 1) for this study. Restored (R1) lakes are featured with a clear boundary, a tree line on lake shores, and are sheltered from disturbance by fisherman boats while unmanaged (R2) lakes are directly connected to natural streams, lacked of any tree line and may be disturbed by passing fisherman boats. The selected lakes were not distant apart, lie within 7 km radius, and shared the same environmental conditions as described earlier.

We then installed four digital video recording cameras (STP110WIP by YeshiYing), 50 m apart from the lake shore, on the top of 50 m high steel towers, to ensure the perspective covering of each sub‐lake (Figure 1). The cameras were equipped with remote moveable devices for left‐right and up‐down movements to position the cameras for recording habitat selection events. We recorded the habitat selection events from 15th Oct to 15th March during 2016–17 and 2017–18 wintering seasons. The recording duration was set from 6 a.m. in the dawn to 6–7 p.m. in the dusk, depending upon the light conditions which vary during this period. Video recording was stopped when the species identification was difficult to carry out in the darkness.

#### 2.2.2 Quantifying habitat selection and habitat identification using video records

We analyzed more than 8,640 hr of video footages (that equals watching a yearlong movie), and filtered the best quality videos with “desired” habitat selection events by the targeted species (Table 1). We define “desired habitat” as “a portion of the sub‐lake devoted to a particular activity of individuals, is suitable for a species when it contains all resources needed for a given activity in sufficient quantity (e.g., food while foraging and roosting sites when resting).” In order to obtain data from each selection event, we waited for the time when species richness and their population density were highest and consistent for at least one hour. In case there are more than one selection events per site, we considered only one selection event per day with highest species richness. During every selection event, we zoomed into the scene to identify the species and counted their numbers distributed between five habitat types: deep water (DW), shallow water (SW), mudflat (MF), grassland (GL) and bare ground (BG). Intermingling of deep water and shallow water habitats was validated in each video using color calibrated PVC pipes (easily readable by digital cameras) which we deployed to record daily water depth. We define deep water habitat as “the portion of lake area with >30 cm water depth”; shallow water as “portion of lake area with 5 cm to 30 cm water depth”; mudflat as “portion of lake area exposed as fen and devoid of any vegetation”; and grassland as “portion of lake area with sparse or complete cover of wet meadows” while bare ground was constituted by stone piles and areas devoid of water and any vegetation. We also validated waterbirds identification through consistent site surveys (2 surveys per site per week) during the study period, using boats.

#### 2.2.3 Identifying waterbirds activities

With the habitat selection event of at least one‐hour duration in video records, we distinguished activities as key behaviors for habitat selection and essential for species survival at wintering grounds (i.e., foraging and roosting) and behaviors as short‐term inter‐ and intra‐species interactions (i.e., aggression, competition, and courtship) during foraging and roosting. We define competition “a behavior when two or more individuals of a species; or individuals of different species aggregated at same physical location (i.e., same portion of the microhabitat) for either foraging or roosting,” while aggression was “the behavior when any of the individual interacted aggressively to overcome this competition.” Thus, for each species in each habitat type, abundance‐based data for foraging and roosting activities were collected while events of inter‐ and intra‐species interactive behaviors were also recorded and studied as abundance (no. of individuals with interactive behaviors) during foraging or roosting (Box 1).

### 2.3 Building social‐behavioral association network (SBAN)

**FIGURE 2:**
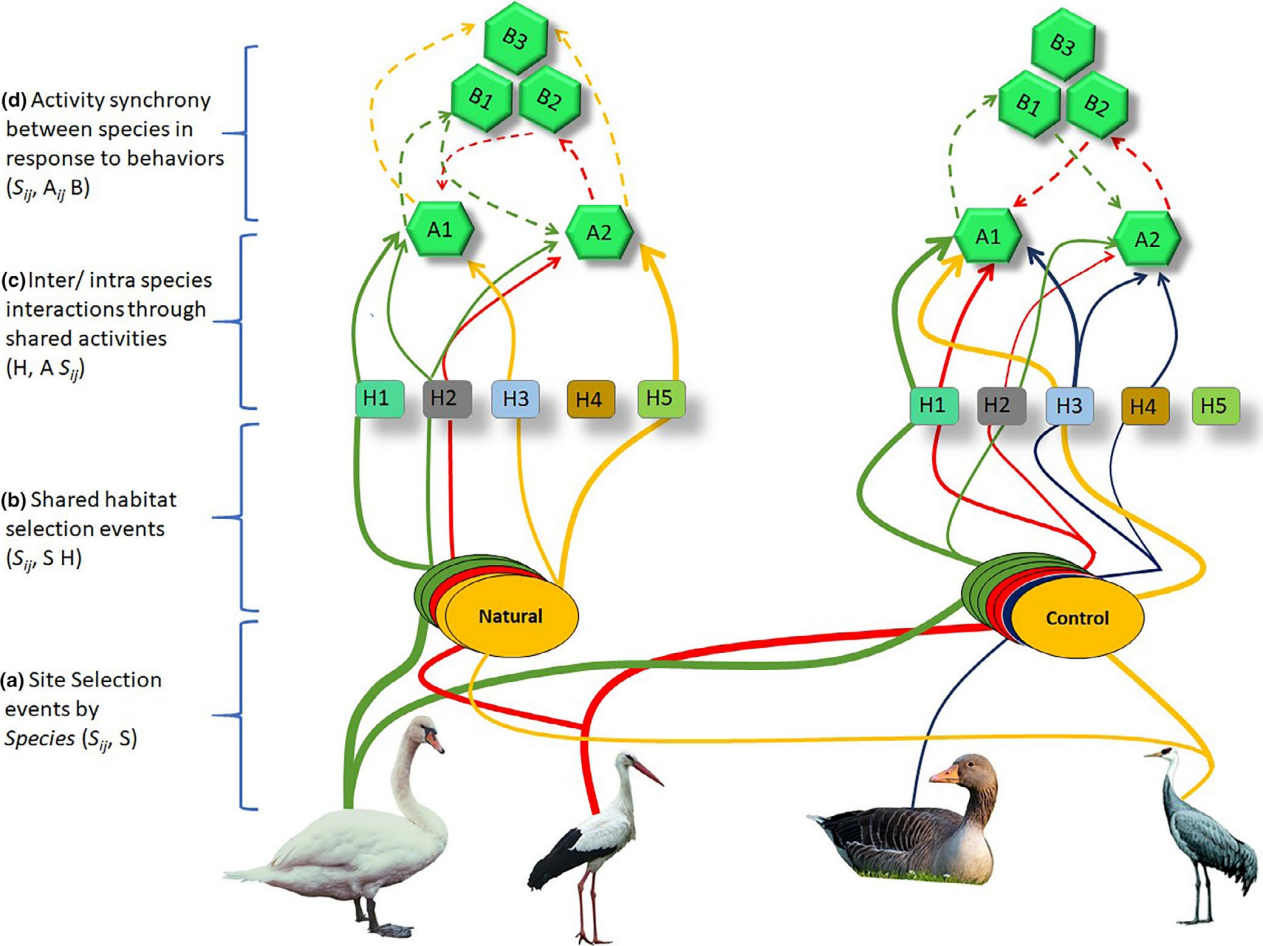
Social‐behavioral association network. The example shows how a community of four co‐occurring species occupies two sites (S). Species select habitat types (H) for given activities (A) and switch between activities in response to some behavior (B). Replications of colored stacks represent the frequency of visits. Solid edges link the species movements between sites and habitats where species freely move or select some site or habitat at multiple times. Dotted edges indicate the activity synchrony in response to some behavior (competition or aggression) that drive other species to switch between different activities while edge width determines the population density moving between one node and another within the network. See text for description of a–d

We constructed a social‐behavioral association network for R1 and R2 using the abundance‐based activity data. This network includes three parts describing individual species identity, distribution in shared space (i.e., microhabitat), and activity to be performed in a particular position in a shared time. Note that prior to the habitat selection event, there is a site selection event playing its part, which the data do not allow us to explore. Together with site selection, the social‐behavioral association network model has four bipartite projections. The first bipartite is a (*S_ij_*, S) projection which aggregates species‐site selection in shared environment (Figure 2a). This projection connects sites (S) on the basis of shared species (*S_ij_*). Note that first bipartite projection was not studied. The second bipartite projection is shared habitat use network within same site (*S_ij_*, S H). It connects species (*S_ij_*) that visit the same habitat (H) (Figure 2b) in shared space (i.e., S). The third bipartite projection is an intra‐/inter‐species interaction through shared activities (H, A *S_ij_*; Figure 2c) where individuals of species (*S_ij_*) interact with one another through presence in the same “aggregation” showing the robustness of habitat (H) for shared activities (A *S_ij_*). The fourth bipartite projection is (*S_ij_*, A*_ij_* B) where species (*S_ij_*) switch between activities (A*_ij_*) in response to a short period behavior (B) (Figure 2d).

#### 2.3.1 Defining nodes and edges in social‐behavioral association network

The two primary aspects of networks are a multitude of separate entities (nodes) and the connections between them (edges). Consider the habitat association of two species, *S*_1_ and *S*_2_, denoted *X*_1 _= {*H*, *A*|*S* = *S*_1_} and *X*_2_ = {*H*, *A*|*S* = *S*_2_}, where *X*_1_ and *X*_2_ are the set of activities in space performed by species *S*_1_ and species *S*_2_, respectively. The intersection of these trajectories, *X*_1_ ∩ *X*_2_ contains all points in space where species *S*_1_ and *S*_2_ co‐occur with their complete set of direct interactions. Because *X*_1_ and *X*_2_ are conditioned on specific individual species, the network produced by them retains species‐specific information in shared space, forming its nodes. Restructuring the conditional term within the habitat association network changes the network projection. For example, if $Y_{1}=\left\{S|H=H_{1}\right\}$ and $Y_{2}=\left\{S|H=H_{2}\right\}$, then the intersection *Y*_1_ ∩ *Y*_2_ is the set of species who visited both *H*_1_ and *H*_2_. A network based on this condition retains information about specific habitat patch use, which forms its nodes. Edges link the individuals of a species *S* who visited habitat patch *H*, but this intersection lacks the information on activity performed by the species. For activity, we reconfigure the network. Given that $Z_{1}=\left\{S,H|A=A_{1}\right\}$ and $Z_{2}=\left\{S,H|A=A_{2}\right\}$ then the intersection *Z*_1_ ∩ *Z*_2_ denotes that the species performed activity A_1_ and A_2_ within the same habitat, forming its nodes.

**FIGURE 3:**
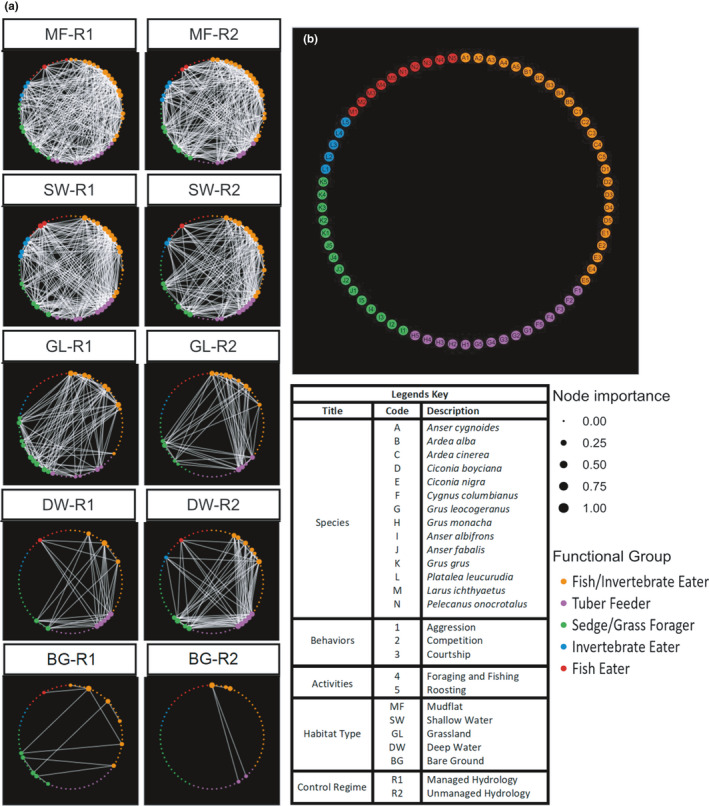
(a) The undirected social‐behavioral association network of wintering waterbirds in managed (left) and unmanaged (right) sites, where species activities and behaviors (5 nodes per species) are connected through edges over five habitat types. The node size is proportional to the connection importance between species and activities. Varying node size with number of edges it has gives rise to vulnerability of the species for a given network. (b) Codes assigned to species for activities and behaviors being performed in every selection event

The network of these nodes connected through edges forms a “real set of interactions” offering wintering waterbirds community dynamics in a shared environment. We created five different SBAN networks (Figure 3a), one for each habitat; with five separate nodes (*n* = 5) for each species (*N* = 14: A–N; A_1,_ A_2_, A_3_, A_4_, A_5_ …. N_1_, N_2_, N_3_, N_4_, N_5_), that represent two activities (foraging and roosting) and three behaviors (competition, aggression and courtship). Each node possesses attributes of the species identity, foraging guild and activity or behavior (Figure 3b). Pairwise observations were converted into edges to populate the adjacency matrix for interactions that represent how two nodes relate to each other and can be used to describe how they associate or interact for social relationships (Castles et al., 2014) in SBAN network.

### 2.4 Assigning node importance and edge weight

Assigning importance to any given species in mutualistic networks is a key task when evaluating species rank over habitat types. Using the *corSparse* function from package “*qlcMatrix*” (Cysouw) in R (R Core Team, 2016), we computed the Pearson correlation (−1 to 1) of activity‐based abundance data and used its absolute values as importance between the columns of sparse matrix to make undirected networking plots utilizing PageRank™ (Csardi & Nepusz, 2006; Heath, 2018). Species network constructed using package “*ggnetwork*” (Briatte, 2016), where nodes were arranged by their relative order of importance, thereby resulted in a highly packed matrix network. We assumed negative Pearson correlation values as zero to simplify the network plots because negative correlation was just seen in one habitat type (BL‐R1). For each habitat type, we made use of hub centrality to rank the species importance (keystones) in the network system.

For interactions (edges) we used PageRank™ algorithm (Bryan & Leise, 2006) to rank the important species (***S***) over habitat (**H**) for activities (**A**) to be performed. For details of the algorithm, please refer to (Bryan & Leise, 2006). In brief, the measure for node importance is the maximum number of edges it connects and range between 0 and 1, where 0 indicates no connection between nodes (less important), and 1 indicates maximum pairing in their respective network system (Croft et al., 2008, 2009; Farine & Whitehead, 2015). Species may move from one habitat type to another at a site to perform or switch between activities. This movement between habitats results in the mutual interactions between species. The problem is similar to that of ranking web pages. Thus, we define a species as important if it interacts (directly or indirectly) with other species that are in turn important. Since species can randomly switch between habitats in the given site, this habitat selection becomes complex and primitive.

#### 2.4.1 Thresholding edges for important nodes and interaction preference scores

Edges carry information on social structure in the network, and filtering networks by thresholding edge weight offers information on attributes of the nodes that they connect (Farine, 2014; Franks et al., 2010). We have set a proportional value of 0.05 (on Pearson correlation values) as a threshold to filter the connectivity matrix to increase the readability of networking plots: all other entries below the threshold were set to “0” and the links will not exist. We then applied an arbitrary cutoff of 0.4 on computed Pearson correlation values and applied PageRank™ to simplify the network density and extracted the information on important interactions between species. When correlation value was higher than 0.4, connections (edges) were included in the network (Mukaka, 2012).

#### 2.4.2 Species interaction preference scores (SIPS) and behavior interaction preference scores (BIPS)

We grouped species according to their trophic guild for the valuable topological information that we leverage to focus their interactions and divided the procedure into two ways. First, the total number of events on each *S*, H, and A was drawn from an unconditional distribution of occupancy in all sites, that is, frequency distributions when each species was observed at some habitat in a site. Second, what activities each species was performing while sharing habitat. Connections were drawn to link individual species to locations of habitat in a site, where each connection bears a tag that indicates when each *S*, H and A pairing occurred (see Figure 2). These spatio‐behavioral metrics describe specific margins of the (*S*, H, and A) array that can inform the set of possible interaction configurations within the social‐behavioral association network (Farine & Whitehead, 2015; Manlove et al., 2018). These edge configurations enabled us to calculate the degree of the networks.

We followed (Wey et al., 2008) for describing species and behavioral interactions. We divided these interactions into species interaction preference scores (SIPS) and behavior interaction preference scores (BIPS) within each habitat type, thereby providing a quantitative measurement of the effects of hydrological regime on species behaviors and hub species role at different habitat types. SIPS define if there exists some interaction between species while BIPS defines how behaviorally different a species or different species interacted. Further, SIPS were distinguished for intra‐ and inter‐species interactions; and BIPS were divided into intra‐ and inter‐behavior interactions (Appendix S1).

### 2.5 SBAN networks comparison using two‐way analysis of variance (ANOVA)

We used analysis of variance (ANOVA) to test the difference of the SBAN characteristics (i.e., number of nodes, number of edges, network density, and SIPS and BIPS) between R1 and R2. Following a significant ANOVA, we used LSD (least significant difference) for post hoc pairwise comparisons. LSD is found very effective and well suited for detecting true difference in the means after a significant ANOVA at 5% significance level (Brown & Behrmann, 2017; Fraiman & Fraiman, 2018; Mason et al., 2003).

## 3 RESULTS

### 3.1 Social‐behavioral association networks (SBAN) in lakes with distinct hydrology

Figure 3a shows the social‐behavioral association network built for the wintering migratory waterbirds community in the managed (R1) and unmanaged lakes (R2). The networks formed in sites with different hydrological regimes displayed great difference in all measured attributes including number of nodes and edges, edge density, and SIPS and BIPS (Figure 3a, Table 2). Surprisingly, compared with unmanaged sites, the sites with controlled hydrology had more species, more active nodes and edges, higher edge density, and BIPS and SIPS but differences were not statistically significantly different at treatment level (*p = *.296; Table 3).

**TABLE 2:** Summary on the attributes of the social‐behavioral association network for R1 (managed sites) and R2 (unmanaged sites)

Regime	Habitat	No. of species (*n*)	Active nodes (*V*)	No. of Edges (*N*)	Density (*d*) %	SIPS		BIPS		Hub species
						Intra‐*spp*	Inter‐*spp*	Intra‐*beh*	Inter‐*beh*	
R1	BG	8	12	78	2.7	5	73	38	40	*A. albifrons, A. fabalis, C. nigra*
	DW	6	11	66	2.3	12	54	14	52	*C. columbianus*
	GL	10	23	253	10.5	20	233	77	176	*A. albifrons*
	SW	13	33	496	21.9	36	460	127	369	*C. columbianus*
	MF	14	40	780	32.3	46	734	200	580	*A. alba, A. cinerea, C. nigra*
R2	BG	3	6	15	0.6	4	11	3	12	*A. cygnoides*
	DW	6	17	136	5.6	22	114	28	108	*C. columbianus*
	GL	6	17	136	5.6	21	115	28	108	*A. fabalis, A. alba*
	SW	11	27	378	14.5	30	348	95	283	*C. columbianus*
	MF	11	33	528	21.9	38	490	135	393	*A. fabalis, P. leucorodia*

**TABLE 3:** Summary on tests of between‐subjects’ effects for SBAN network attributes of managed (R1) and unmanaged (R2) sites

Source	Type III Sum of squares	*df*	Mean square	*F*	*p*‐Value
Corrected Model	679,722.129^a^	9	75,524.7	2.994	.004
Intercept	1,017,659	1	1,017,659	40.346	.000
Sites	27,919.1	1	27,919.1	1.107	.296
Habitat	619,593	4	154,898	6.141	.000
Sites × Habitat	32,210.4	4	8,052.61	0.319	.864
Error	1,765,648	70	25,223.6		
Total	3,463,030	80			
Corrected total	2,445,371	79			

^a^ *R* squared = .278 (Adjusted *R* Squared = .185).

For different habitats within a site, mudflats had far more complex network followed by shallow waters and grasslands while bare grounds and deep waters had the simplest networks, no matter what the hydrological regime was (Figure 3a, Table 2). For example, we recorded 14 species in mudflat at R1 site, which formed a network of 40 nodes connected through 780 edges, resulting in the highest density score of 32.3 (Table 2). On the other end, we only recorded 3 species in bare ground at R2 site without hydrological control, where the waterbirds formed the simplest social‐behavioral network with six nodes linked by 15 edges, with lowest edge density of 0.6 (Table 2). Differences in these network attributes tested by two‐way analysis of variance (ANOVA) at 0.05 significance level were highly significant (*p* = .000; Table 3).

### 3.2 Keystone species and their dominances over habitat types

**FIGURE 4:**
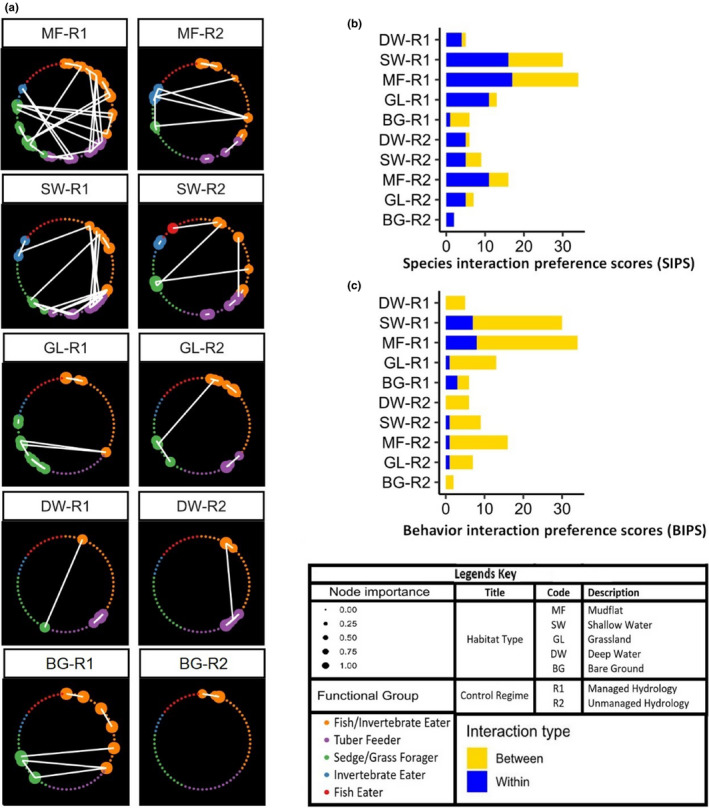
(a) The simplified network using an arbitrary cutoff of 0.4 in Pearson correlation to highlight the important connections and keystone species where species are ranked according to their importance (hub centrality) in the community at each habitat type. (b) Inter‐ and intra‐species interaction preference scores. (c) Inter‐ and intra‐behavioral interaction preference scores (BIPS)

Species changes their role at different habitats (Table 2). Moreover, besides deep waters and shallow waters in both regimes, other habitats were far dissimilar in keystone species composition and their dominances (Table 2; Figure 4a). In deep water and shallow water habitats, *C. columbianus* was the keystone species, however, its dominancy was challenged by *A. alba* in deep waters of R2. *Ardea alba*, *Ardea cinerea* and *Ciconia nigra* were keystone in mudflats of R1 while *Platalea leucorodea* and *Anser fabalis* were keystone species at R2 mudflat habitats. *Anser fabalis* and *Ardea alba* were keystones at grasslands of R2 while *Anser albifrons* was keystone at R1 grasslands. *Anser cygnoides* in R2 bare ground and *Anser albifrons*, *Anser fabalis* and *Ciconia nigra* at R1 bare grounds were keystones.

### 3.3 Species interaction preference scores and behavior interaction preference scores

Except bare grounds at R2, all other networks had inter‐species interactions, indicating the prevalent competitions for available resources in the wintering grounds. In the managed sites, mudflats had the highest value of inter‐SIPS (734), followed by shallow water (460), grassland (233), bare grounds (73), and deep waters (54). On the other hand, mudflats (46), followed by shallow water (36), grassland (20), deep water (12), and bare grounds (5) had the lowest value of intra‐SIPS. Intra‐SIPS values in the uncontrolled sites were also highest for mudflats (490) followed by shallow water (348), grassland (115), deep water (114) and bare ground (11), while intra‐SIPS values for mudflats was 38 followed by shallow waters (30), deep water (22), grassland (21) and bare grounds (4) (Figure 4b, Table 2).

For BIPS, the indicator of activity synchrony, nearly all habitats have behavioral interaction (intra‐ & inter‐) in both regimes (Table 2), suggesting there was activity synchrony between species. In managed habitats (R1), highest activity synchrony (inter‐BIPS) was in mudflats, followed by shallow water, grassland, deep waters and bare grounds with 580, 369, 176, 52 and 40 respectively. The lowest value for this activity synchrony was seen in bare ground (12) of unmanaged sites (R2), followed by deep water, grassland, shallow water and mudflats having 108, 108, 283 and 293 scores respectively (Figure 4c, Table 2).

### 3.4 Species SBAN networks were far dissimilar in different habitat types

Some of the habitats (both in R1 and R2) were significantly different since the *P*‐value was found to be <0.05. The mean comparison between the habitats revealed that BG and DW were not different from each other but significantly different from SW and MF, GL was significantly different from MF at 5% level of significance while MF and SW were not different from each other at 5% level of significance, supported our results (Appendix S2). These assertions were further supported by dramatic differences in keystone species composition at different habitat types, as previously described.

## 4 DISCUSSION

In this paper, we presented a framework of using SNA to graphically visualize and quantitatively describe the patterns of associations or interactions data obtained through video recording cameras for wintering waterbirds communities. We compared animal social‐behavioral networks for the bird communities using sites with distinct management (i.e., managed and unmanaged hydrology). We demonstrated that the SNA approach is a powerful tool to link individual species behavior with community‐level patterns and processes (Krause et al., 2015). We found the dramatic differences in network structure, such as density and assortativity between communities occupying sites with distinct hydrological regimes. Despite the striking dissimilarity in hub species, the inter‐ and intra‐species interactions, use of habitat patches might be determined by phylogeny (Balasubramaniam et al., 2018), phenotypes (McDonald et al., 2017) and species behavior (Stonehouse et al., 2015).

**FIGURE 5:**
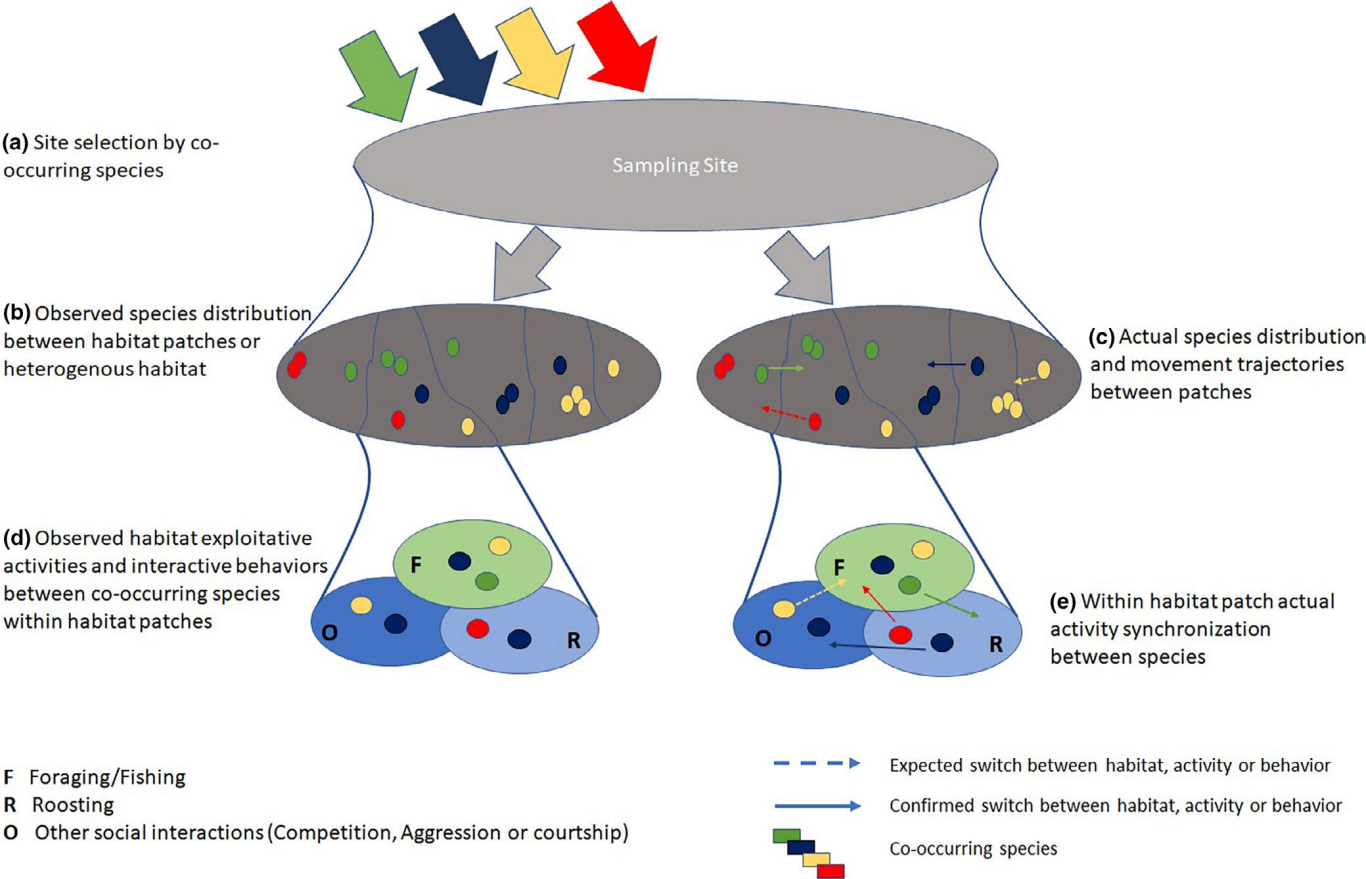
Animal's movement depend on the environment devoted to particular activities of individual species when it contains all resources in sufficient quantity for a selection event to occur (a) where species distribute in the shared space for habitat use and preferences (b), these habitat use mechanisms allow animals to exploit habitat patches for given activities (c), these exploitation‐based movements between habitat patches offer contact patterns between species (d), and these interactions among co‐occurring species cause in many cases activity synchrony to avoid potential competition between species (e)

In a heterogenous habitat, co‐occurring species in a community require to select specific site in a shared environment (Figure 5a,b). Numerous interactions and social connections influence individual species distribution between habitat patches (Figure 5c). Such distribution patterns could lead to the exhibition of aggression, competition or dominance behaviors (Fretwell, 1969; Smith & Metcalfe, 1997) as a result of limiting resources in the most suitable environment (Marra, 2000). Consequently, animals divide their distribution in shared space and synchronize time between multiple activities, especially foraging and resting (Halle & Stenseth, 2012) (Figure 5d). In gregarious species, aggregation size preferences predict within and between species contact preferences (Manlove et al., 2018). These contacts induce pressure on other species to drive different activity to avoid potential resource competition thus segregating the temporal niche and minimizing the risks of competition (Figure 5e) in shared space.

### 4.1 Species' dominance in different habitat types

Stability and robustness assessment in complex ecosystems is a basic problem in conservation ecology (Dunne et al., 2002, 2004). The loss of an individual “keystone or hub” species may induce effects on other important species, resulting in re‐configuration of the entire network structure. Thus, the relative “importance” of a given species within a network can be viewed as a function of the integrity and robustness within the network.

In shallow and deep waters, *C. columbianus* was hub of the network in both regimes and dominated over other species such as *A. cygnoides, A. alba, G. leucogeranus, A. cinerea, A. albifron,* and *Larus* spp, among which *C. columbianus* is the heaviest (averaged at 6.4 kg, (Wilman et al., 2014). Our findings provide support for a key prediction that large body size helps species acquire high dominance ranks over occupied habitats. This dominance pattern was found across a range of animals ((Haley et al., 1994; Neumann et al., 2011; Pelletier & Festa‐Bianchet, 2006; Pusey et al., 2005).

The single species dominance was not observed in other habitat types. In mudflats, species with the same foraging guild and similar body size, such as herons (*A. alba*, *A. cinerea*) and storks (*C. nigra*), were the keystone. Such distribution patterns in gregarious species, led to the exhibition of aggression or competition behaviors between dominant species, especially when body size allow some species a greater access to limited resources. At these habitats, the aggregation size predicts within and between species contact preferences (Fretwell, 1969; Manlove et al., 2018; Smith & Metcalfe, 1997). These contacts induce pressure on other species to synchronize different activity to avoid potential resource competition thus segregating the temporal niche and minimizing the risks of competition in shared space (Marra, 2000), supported by our findings.

Additional morphological characteristics and intransitivity for coexistence that explain shifts in dominance, beyond body mass toward the root of the phylogeny (e.g., families) deviate from the overall mass‐to‐dominance relationships (Allesina & Levine, 2011; Laird & Schamp, 2006, 2008; Miller et al., 2017). This has been previously demonstrated for plant competition in laboratory (Keddy & Shipley, 1989; Kerr et al., 2002; Soliveres et al., 2015). However, tests of this idea in animals are rare (Levine et al., 2017). For instance, *A. albifrons* and *P. leucorodia*, even though these two species have different feeding guild, both species were dominant in mudflats of R2, this apparently mutual dominance between these species may simply be a function of resource competition, same as in case with grassland at R2, where *A. fabalis* and *A. alba* were dominant.

### 4.2 Inter‐ and intra‐species interaction and activity synchrony

Measuring important habitat features (food and cover) in meaningful ways for management is often difficult, labor intensive and costly (Henschel & Ray, 2003). Consequently, wildlife managers often rely on surrogate variables, such as frequency of habitat selection (as described in previous section) and interaction profiles, which are relatively easy to measure in the field or can be interpreted from databases (Kays et al., 2015; Stonehouse et al., 2015; Wilmers et al., 2015).

With the realized social‐behavioral association network, species interaction preference scores (SIPS) for interaction (resource competition between species) and intra‐species interaction (resource competition between individuals of a species) can be easily calculated. The SIPS can also be used to assess habitat quality. For example, deep waters at managed lakes were the most suitable habitat for *C. columbianus* while bare grounds at unmanaged lakes were best for *A. cygnoides* (Table 2; Figure 4a). Grasslands at unmanaged lakes were more suitable with lesser amount of intra‐species interaction than at managed lakes while shallow water at managed lakes were better habitats with reduced intra‐species interactions. High (absolute) interaction scores in mudflats and shallow waters at both R1 and R2 sites suggested that while many water bird species preferred these habitats, spatial competition (especially between hub species) was also strong in these habitat types.

Numerical measures of synchrony are complicated to define, identify and deal with. In general, it increases when many species in subnetworks exhibit broad dominance hierarchy (Shizuka & McDonald, 2012, 2015). Linking these dominance hierarchies to behavioral interactions between species gives insights for activity synchrony in subnetworks (habitat patches) among co‐occurring species (Mukherjee et al., 2009; Rasool et al., 2015; Suselbeek et al., 2014), which in our case are defined as BIPS (behavioral interaction preference scores).

At both managed (R1) and unmanaged lakes (R2), BIPS were linked to resource competition and aggression behavior. Inter‐species behavioral interactions of *C*. *columbianus* in deep waters and shallow waters resulted in avoidance of these habitats by Swan geese as foraging grounds (but they used the habitats for roosting). As described earlier, this might be due to the function of body size and aggression. Grassland habitats gave insight to the increased BIP score possibly because of competition between species, therefore driving other species to avoid or switch their activities to minimize the potential competition. Based on frequency of site selection events, it can be said that the species (especially *G. Leucogeranus, G. monacha, C. boyciana, C. nigra and A. albifrons*) somehow avoided unmanaged lakes (R2) while the species selecting these lakes were thereby friendly to share the habitats for given activities.

### 4.3 Influence of hydrological regimes on habitat selection, interactions and network structure

Reliable information about the quality of habitat features is critical to predict the performance of occupying animals for their conservation (Beck et al., 2014). Studies on animal behaviors and interactions among individual species can provide answers to many questions in wildlife ecology and conservation. Using social‐behavioral association network approach, we found that there were more inter‐ and intra‐species interactions in managed lakes than unmanaged lakes (Figure 4a). This finding was further supported by the total number of species that selected managed lakes over unmanaged lakes (Table 2). Generally, for each habitat type, more species were recorded in managed lakes than in unmanaged ones. Out of the total 14 species that we focused in this study, all of them were observed using the mudflats and 13 were recorded in shallow waters at the managed lakes. However, in unmanaged lakes, we found only eleven species using these habitats. Moreover, ten species selected grassland habitat patches at managed lakes while just six species selected grassland patches of unmanaged lakes. In bare grounds, eight species in managed and three species in unmanaged lakes were found. These results provided a clear indication of better habitat quality at managed lakes. As animals select habitat at a variety of spatial and temporal scales, this information becomes relevant to the scale of management and reflects the functional value of the habitat for population persistence (Godvik et al., 2009; Silveira et al., 2003; Wegge et al., 2004).

## 5 CONCLUSION

In this study, we introduced a novel approach of building abundance‐based social‐behavioral association network of species using video footages. We demonstrated the applicability of this approach in wildlife management and conservation by a cased study comparing wintering waterbirds networks in managed and unmanaged lakes. The results of social network analysis suggested that the number of species, the number of network nodes and edges, edge density, and intra‐ and inter‐species interactions were all dramatically higher in managed lakes than in unmanaged ones. These findings indicated a better habitat quality in managed lakes. Nevertheless, the higher species richness and abundance also resulted in more intensive intra‐ and inter‐species competition for limited resources in the managed lakes. Our approach is likely to provide a practical use for ecosystem management and biodiversity conservation, where there is a need to make decisions on what aspects of ecosystems conservation and management should be focused.

## CONFLICT OF INTEREST

No conflict of interest has been declared by the author(s).

## AUTHOR CONTRIBUTION

**Muhammad Awais Rasool:** Conceptualization (lead); Data curation (lead); Formal analysis (lead); Investigation (lead); Methodology (lead); Project administration (equal); Software (equal); Validation (lead); Visualization (lead); Writing‐original draft (lead); Writing‐review & editing (lead). **Xiaobo Zhang:** Investigation (supporting); Project administration (equal); Resources (equal); Writing‐review & editing (supporting). **Muhammad Azhar Hassan:** Data curation (equal); Formal analysis (equal); Software (equal); Writing‐review & editing (equal). **Tanveer Hussain:** Methodology (equal); Validation (equal); Writing‐review & editing (equal). **Cai Lu:** Data curation (equal); Funding acquisition (equal); Project administration (equal); Resources (equal); Writing‐review & editing (equal). **Qing Zeng:** Conceptualization (equal); Methodology (equal); Validation (equal); Writing‐review & editing (equal). **Boyong Peng:** Project administration (equal); Resources (equal); Validation (equal). **Li Wen:** Methodology (equal); Software (equal); Supervision (equal); Writing‐review & editing (equal). **Guangchun Lei:** Conceptualization (equal); Funding acquisition (lead); Project administration (lead); Supervision (lead); Validation (equal); Writing‐review & editing (equal).

## Supporting information

Appendix S1

Appendix S2

Appendix S3

Appendix S4

Appendix S5

## Data Availability

The data used to construct SBAN are available at https://doi.org/10.5061/dryad.vmcvdncrt.
