# Supplementary material for: Construct social‐behavioral association network to study management impact on waterbirds community ecology using digital video recording cameras
Source: Ecol Evol. 2021 Feb 1;11(5):2321–35. doi: 10.1002/ece3.7200 (PMC7920787; doi:10.1002/ece3.7200)
Supplement: Supplementary file 1 — Appendix S1 [file ECE3-11-2321-s001.docx]

### Species interaction preference scores (SIPS) and behavior interaction preference scores (BIPS)

### Intra- and Inter-species interactions

For SIPS, each species (A-N) in studied network is composed of five vertices (as previously described). A multi-subgraph having each vertex equals to adjacency matrix Ai*j (A5*5 activities) provides information on frequency of intra- and inter-species interactions as given in equation 1 and 2, respectively, to inform whether a species has interacted with other species or not.

(1)

where, $\deg(A)^{\partial}$ denote intra degree of nodes of A, $\sum\deg(A...N)^{\partial}$is the intra degree of graph for all species (A – N), $k_{i}$ donates degree of each node with self-adjacent edges in each species, and *n* is equal to 5 behaviors.

$\sum\deg(A...N)^{\gamma}=\sum\deg(A...N)^{tot}-\sum\deg(A...N)^{\partial}$ (2)

where, $\sum\deg(A...N)^{\gamma}$is sum of inter connected nodes degrees for all species: A-N, and $\sum\deg(A...N)^{tot}$ is total summation of degree for whole network graph.

### Intra- and inter-behavior interactions

For BIPS, we have considered multigraph having each vertex equals to adjacency matrix A_i*j_ (A_14*14_ species) depicted information on frequency of intra-behavior interactions by measuring self-looped edges (A-N; eqn. 3) and inter-behavior degree through interconnected edges between 1-5 (eqn. 4). BIPS is the numeric explanation for interaction preference for behavioral synchrony between species.

$\sum\deg(1...5)^{\partial}=\sum_{i=1}^{n} k_{i}(1...5), i=1,2,3,\ldots n$ (3)

where, $\sum\deg(1...5)^{\partial}$ is the summation of intra degree of graph for all behaviors (1 – 5), $k_{i}$ donates the degree of each node with self-looped edges in each behavior, and *n* equals to 14 species.

$\sum\deg(1...5)^{\gamma}=\sum\deg(1...5)^{tot}-\sum\deg(1...5)^{\partial}$ (4)

where $\sum\deg(1...5)^{\gamma}$ sum of inter connected degrees for all vertices (behaviors: 1-5), and $\sum\deg(1...5)^{tot}$ is total summation of degree for whole network graph.
