# Supplementary material for: Construct social‐behavioral association network to study management impact on waterbirds community ecology using digital video recording cameras
Source: Ecol Evol. 2021 Feb 1;11(5):2321–35. doi: 10.1002/ece3.7200 (PMC7920787; doi:10.1002/ece3.7200)
Supplement: Supplementary file 3 — Appendix S3 [file ECE3-11-2321-s003.docx]

| **Network Scope** | | **Description** | **Notation** | **Edges in the network** |
| --- | --- | --- | --- | --- |
| **a** | Habitat selection | Set of positions used by species | $X_{k}=\left\{\left(\left(H\times A\right)\vert S=S_{k}\right)\right\}$ |   Nodes that occur in proportion to the number of patches used by species *i* that are also used by species |
| **b** | Habitat overlap | Set of species that occupied habitat H*_k_* | $X_{k}=\left\{S\vert H=H_{k}\right\}$ |   Nodes that occur in proportion to the number of species visiting habitat *i* that also visit habitat *j* |
| **c** | Species-Habitat associations | Set of aggregations with *S_k_* as a member | $X_{k}=\left\{\left(\left(H\times A\right)\vert S=S_{k}\right)\right\}$ |   Nodes that occur in proportion to the number of aggregations that include species *i* that also include species *j* |
| **d** | Intra species behavioral associations within a habitat | Set of species present in aggregation (H×A)*_k_* | $X_{k}=\left\{S\vert\left(H\times A\right)=\left(H\times A\right)_{k}\right\}$ | Nodes that occur in proportion to the number of aggregations that include species *i* that also include species *j* |

Social-behavioral association network models in terms of (*S*, H, A). “**||**” indicates magnitude (the count of the size of the set), {} indicates a set fulfilling some condition. ‘ǀ’ is the conditioning bar and “**∩**”is the intersection symbol.
