# Supplementary material for: Construct social‐behavioral association network to study management impact on waterbirds community ecology using digital video recording cameras
Source: Ecol Evol. 2021 Feb 1;11(5):2321–35. doi: 10.1002/ece3.7200 (PMC7920787; doi:10.1002/ece3.7200)
Supplement: Supplementary file 5 — Appendix S5 [file ECE3-11-2321-s005.docx]

| Term | Definition |
| --- | --- |
| Social-behavioral association network | Social-behavioral association network (SBAN) is a network of species in a community where species are interconnected with other co-occurring through behavioral communication, principally to exploit shared resources. |
| Keystone species | A species that plays a crucial role in the way an ecosystem maintains its integrity. Disturbance to keystone species alters the ecosystem structure to degradation in long run. |
| Hub centrality | The capacity of a node (species) to influence and mediate between other nodes (peripheral species) by its virtual connectivity. |
| Activity | Key behavior for habitat selection and essential for species survival at wintering grounds (i.e., foraging and roosting). |
| Behavior | A short-term inter- and intra-species interaction (i.e., aggression, competition and courtship) that may occur while performing resource exploitative activities (i.e., foraging or roosting). |
| Species interaction preference scores (SIPS) | Quantitative measure of any intra- or inter-species interactions during shared activity. |
| Behavioral interaction preference scores (BIPS) | Quantitative measure of intra- or inter- species-specific behavioral interactions on how the species interacted |
| Activity synchrony | The spatio-temporal facilitation behavior between co-occurring species to exploit shared resources in principally to avoid any potential event for competition. |
